# MicroRNA-181c prevents apoptosis by targeting of FAS receptor in Ewing’s sarcoma cells

**DOI:** 10.1186/s12935-018-0536-9

**Published:** 2018-03-12

**Authors:** Masanori Kawano, Kazuhiro Tanaka, Ichiro Itonaga, Tatsuya Iwasaki, Hiroshi Tsumura

**Affiliations:** Department of Orthopaedic Surgery, Faculty of Medicine, Oita University, Oita, 879-5593 Japan

**Keywords:** Ewing’s sarcoma, MicroRNA, miR-181c, FAS

## Abstract

**Background:**

MicroRNAs (miRNAs) are endogenous, small non-coding RNAs that play important roles in multiple biological processes. Here, we show that miRNAs play an important function in the down-regulation of FAS expression in Ewing’s sarcoma (ES) cells.

**Methods:**

To identify and characterize possible oncogenic factors in ES, we employed a microarray-based approach to profile the changes in the expression of miRNAs and their target mRNAs in five ES cell lines and human mesenchymal stem cells (hMSCs).

**Results:**

MiRNA, miR-181c, was significantly up-regulated, whereas FAS receptor expression was significantly down-regulated in all tested ES cells compared with hMSCs. Introducing anti-miR-181c into ES cell lines resulted in an increased expression of FAS2. Additionally, anti-miR-181c prohibited cell growth and cell cycle progression in ES cells. Anti-miR-181c also promoted apoptosis in ES cells. Furthermore, the down-regulation of miR-181c in ES cells significantly suppressed tumor growth in vivo.

**Conclusions:**

These results suggest that unregulated expression of miR-181c could contribute to ES by targeting FAS. Reduction of miR181c increased expression of FAS. This proves that retardation of cell cycle progression removes apoptosis resistance, thereby repressing the growth of Ewing sarcoma. Since FAS signaling is involved in regulation of apoptosis and tumor proliferation, our findings might contribute to new therapeutic targets for ES.

## Background

Ewing’s sarcoma (ES) is the second most frequent primary malignant bone tumor in children and adolescents. ES is characterized by specific translocations resulting in the fusing the EWS gene with different members of the ETS transcription family, the most frequent is being the EWS-FLI-1 fusion [1]. Over the past two decades, there has been great efforts have been made to elucidate the underlying mechanisms of ES development over the past two decades, and to identify novel therapeutic targets for ES patients [2]. However, survival estimates remain miserable for patients who present with overt metastatic disease or who relapse following initial therapy, survival estimates remain miserable.

MicroRNAs (miRNAs) are a large family of non-coding single-stranded RNAs of 18–25 nucleotides that play a role in post-transcriptional regulation. miRNAs have been shown to be involved in many cellular processes, and are implicated including in cancer as either tumor suppressors or oncogenes [3]. By binding of the miRNA to a partially homologous sequence mostly usually located within the 3′untranslated region (UTR) of the target mRNA a transcript, it can either block its translation of the target mRNA translation or lead to its degradation. And miRNAs play important roles in the regulation of gene expression both in normal tissues and as well as in disease pathogenesis [4–6].

FAS, also known as (also termed APO-1, CD95, tumor necrosis factor receptor superfamily member 6, or TNFRSF6) is a death domain-containing member of the TNF receptor superfamily [7]. Binding to Fas by its physiological ligand, FasL, triggers receptor trimerization, followed by formation of the death-inducing signaling complex (DISC), and subsequent apoptosis [8]. Several reports have demonstrated that FAS is involved in cell apoptosis in human cancer [9, 10].

In the present study, we conducted explored genome wide whole genome array expression analysis of both miRNAs and mRNAs in human mesenchymal stem cells (hMSCs) and five human ES cell lines. The results showed that the expression of miR-181c was increased in all five ES cell lines, whereas that of FAS was repressed in all five ES cell lines. We hypothesized that the effect of FAS in ES cells might be mediated, at least in part, via miR-181c, directly or indirectly. The aim of our study is to evaluate whether the expression of FAS is repressed by miR-181c and the pathway could play a role in malignancy in ES cells.

## Methods and materials

### Cell lines and reagents

Human MSC (hMSCs) was obtained from Takara Biotechnology. Human Ewing sarcoma cells (SK-N-MC, RD-ES, SK-ES-1 and SCCH) were purchased from Japanese Collection of Research Bioresources (Tokyo, Japan). WE-68, a human ES cell line, was generously provided by Prof. Frans van Valen (Westfalische-Wilhelms University, Munster, Germany). High glucose-Dulbecco’s modified eagle medium (DMEM), RPMI 1640 medium, minimal essential medium (MEM), and fetal bovine serum (FBS) were purchased from Invitrogen (Carlsbad, CA, USA). Mesenchymal Stem Cell (MSC) Basal Medium, Chemically Defined (MSCBM-CD) and MSCGM-CD SingleQuats were obtained from Takara Biotechnology (Otsu, Japan). RNeasy kit and miRNeasy Mini kit were obtained from Qiagen, (Valencia, CA, USA), and TRizol reagent was from Invitrogen. The microRNAs, miR-181c-5p mimic (5′-AACAUUCAACCUGUCGGUGAGU-3′), miR-181c-5p mutant (5′-AUGUAAGUACCUGUCGGUGAGU-3′), hsa-miR-181c inhibitor or negative control (NC) miRNAs were purchased from Invitrogen. A transfection reagent Lipofectamine 2000 and antibiotics-free OptiMEM were also obtained from Invitrogen. Actinomycin D was from Sigma-Aldrich (Tokyo, Japan). FAS expression plasmid (SC321759) was obtained from OriGene Tech. Inc (Iowa, USA). Antibodies produced in rabbits for FAS (#8023), caspase 3 (#9662), cleaved caspase 3 (#9661), caspase 7 (#9492), cleaved caspase 7 (#9491), caspase 8 (#4790), cleaved caspase 8 (#9496), PAR/poly (ADP-ribose) polymerase (PARP) (#9542), cleaved PARP (#9541) and β-Actin (#4970) were purchased from Cell Signaling Technology (Tokyo, Japan). Horseradish peroxidase-conjugated anti-rabbit immunoglobulin G antibodies and ECL Prime system were obtained from GE Healthcare (Tokyo, Japan). Cycletest Plus DNA reagent kit and Annexin V-FITC apoptosis detection kit was obtained from BD Biosciences (Tokyo, Japan). BALB/c nu/nu nude mice were purchase from Kudo (Tosu, Japan). All protocols for animal experiments in the present study were approved by the Ethics Review Committee for Animal Experimentation of Oita University.

### Cell culture

SK-N-MC and RD-ES cells were cultured in DMEM high-glucose medium and SK-ES1 and WE-68 cells were cultured in RPMI 1640 medium. The media were supplemented with 10% FBS and 1% penicillin/streptomycin. SCCH cells were cultivated in MEM medium supplemented with 10% FBS and 0.1 mM non-essential amino acids. hMSCs were maintained in MSCBM-CD supplemented with SingleQuats. The cells were incubated at 37 °C in the incubator chamber supplemented with 5% CO_2_ and passaged when the cells were grown to approximately 70% confluent.

### miRNA and mRNA expression analysis using microarray

miRNA and total RNA were extracted by miRNeasy and RNeasy kits, respectively, from the cells according to the manufacturer’s recommendation. The quality of RNA was confirmed using Bioanalyzer 2100 (Agilent, Santa Clara, CA). An aliquot (1 μg) of small RNA fraction including miRNAs from each of five ES cells and hMSCs was biotin-labeled by FlashTag Biotin HSR Kit (Genisphere, Hatfield, PA) and subjected to miRNA expression array analyses with GeneChip miRNA 3.0 array (Affymetrix, Santa Clara, CA). The array data were quantile normalized, log2-transformed using miRNA QC software (Affymetrix), and analyzed using GeneSpring GX 11.0 (Agilent). For mRNA expression analysis, 1 ng total RNA from each of five ES cells and hMSCs was used to generate double stranded cDNA by reverse transcription, and then used to generate biotinylated cRNA by in vitro transcription using the 3′IVT Express Kit (Affymetrix). The cRNA probes were subjected to hybridization to GeneChip Genome HG U133 Plus 2.0 Array (Affymetrix). GeneSpring GX 11.0 software was used for the array analyses including normalization and filtering (20.0–100.0th‰). The whole experiments were repeated twice. The variant analyses were carried out to determine the significant difference between two groups. Genes indicating twofold or more significant increase or reduction in the expression were listed up. KEGG pathway database (http://www.genome.jp/kegg/pathway.html) was used for the pathway analyses.

### Target prediction of miRNAs

To predict miRNAs target genes, microRNA.org (http://www.microrna.org/), TargetScan 6.0 (http://www.targetscan.org/), Basic Local Alignment Search Tool (BLAST), DIANA tools (http://diana.imis.athena-innovation.gr/DianaTools/), and PicTar (http://pictar.mdc-berlin.de/) were used. The results of the database analyses suggested that FAS was the strongest target of miR-181c.

### Transfection and cell proliferation analysis

Cells (1 × 10^5^) were cultured in 2 ml medium without antibiotics in 6-well plates. miR-181c-5p mimic, miR-181c-5p mutant, anti-miR-181c inhibitor, negative control miRNAs, FAS expression vector and Mock vector were transfected using Lipofectamine 2000. 10 μg/ml of Actinomycin D was used to inhibit the de-novo RNA transcription. The transfected cells were incubated for 48 h and subjected to further analyses. The number of viable cells was counted by TC10 Automated Cell Counter (BioRad). The cell cycle distribution was monitored by propidium iodide (PI) staining using a fluorescence activated cell sorting (FACS) analyzer FACSVerse (BD Biosciences). The number of cells in the cell cycle phases of G0/G1, S, and G2/M were analysed. All experiments were performed in triplicate manner.

### Quantitative RT-PCR

The transfected cells were harvested and lysed using TRizol reagent for the extraction of total RNA. Then cDNA was generated and quantitative real-time PCR (qRT-PCR) was carried out with Light Cycler 480 (Roche). The relative expressions of FAS and GAPDH were calculated by 2-(ΔΔCt) method. The following primers were used; FAS-forward 5′-TGCAGAAGATGTAGATTGTGTGATGA-3′, FAS-reverse 5′-GGGTCCGGGTGCAGTTTATT-3′; GAPDH-forward 5′-CCTCTATGCCAACACAGTGC-3′, GAPDH-reverse 5′-GTACTCCTGCTTGCTGATCC-3′.

### Western blotting

The cellular protein was extracted and an aliquot (15 μg) was applied onto 10% Tris–HCl Criterion precast gel (Biorad). The proteins on the gel were transferred onto PVDF membrane, and reacted with anti-FAS (#8023), caspase 3 (#9662), cleaved caspase 3 (#9661), caspase 7 (#9492), cleaved caspase 7 (#9491), caspase 8 (#4790), cleaved caspase 8 (#9496), PAR/poly (ADP-ribose) polymerase (PARP) (#9542), cleaved PARP (#9541) and β-Actin (#4970) were purchased from Cell Signaling Technology (Tokyo, Japan). The blots were treated with anti-rabbit IgG antibodies and signals were detected using ECL Prime system (GE Healthcare). The quantification of the protein expression was carried out using ImageQuant TL software (GE Healthcare). All experiments were done in triplicate.

### Detection of apoptosis

The cells underwent apoptosis were detected by FACS analysis and western blotting. SK-ES-1 (1 × 10^6^ cells) were cultured and transfected with anti-miR-181c miRNA or FAS expression vector. Forty-eight hours after the transfection, the cells were subjected to analysis using FACSVerse for detection of cell death by the expression of Annexin V. As the positive control of apoptosis, the cells were treated with low dose (5 μg/ml) of doxorubicin. The expression of apoptosis-related proteins, including PARP and cleaved PARP were also analyzed by western blot.

### In vivo experiments using nude mice

SK-ES-1 cells (2 × 10^6^) transfected with anti-miR-181c were suspended in 100 μl normal saline and injected into the gluteal region of BALB/c nu/nu mouse. Total of 20 mice were divided into four groups (5 mice each): (1) untreated control, (2) transfected with control-miRNA, (3) transfected with anti-miR-181c, and (4) transfected with FAS expression vector. The changes in weight of the treated mice and volume of the tumors were monitored for 6 weeks. The volume of the tumor was calculated using the formula of V = (length × width^2^)/2. Then mice were sacrificed and xenografted tumors were removed and subjected to immunohistochemistry. The resected tumors were fixed with 4% formaldehyde, paraffin embedded, sectioned using microtome, and reacted with anti-FAS and cleaved caspase 3 (#9661) antibodies were purchased from Cell Signaling Technology (Tokyo, Japan). The expression of proteins in the section was visualized using DAB and EnVision System (Dako).

### Statistical analysis

Two-tailed Student’s *t* test was carried out for continuous variables. The differences among more than 3 groups were analyzed using ANOVA and Scheffe test. The results were expressed as the mean ± standard deviation (SD), the differences were considered significant when p value were less than 0.05. All statistical analyses were done using SPSS 23.0 software (IBM, Tokyo, Japan).

## Results

### Expression of miR-181c in ES cells

**Fig. 1:**
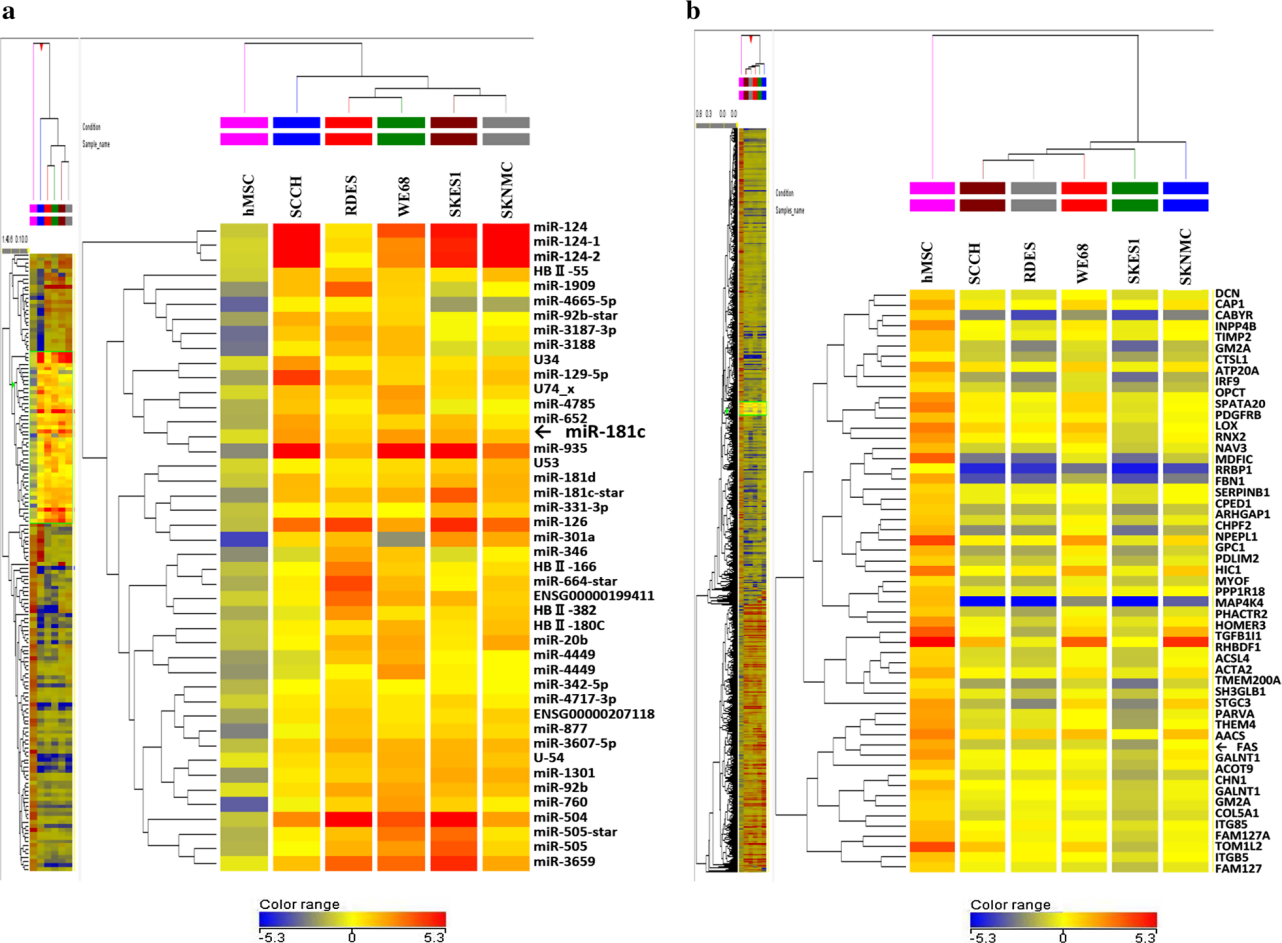
Whole genome array analysis in ES cell lines. **a** miRNA expression in five ES cell lines (SCCH, RDES, WE68, SKES1 and SKNMC) and hMSCs. **b** Heat maps of mRNA expression in ES cells and hMSCs. The color bar shows the relative expression levels; red and blue indicate increase and decrease respectively

Microarray analysis was carried out to determine the expression profiles of miRNAs in ES cell lines. The results demonstrated that 1054 miRNAs in ES cells showed significantly altered expression (more than twofold-change) compared with hMSCs (Fig. 1a). The expressions of 228 miRNAs out of 1054 significantly increased, whereas those of 705 were significantly decreased in all ES cell lines tested. The remaining 121 miRNAs exhibited different expression patterns among five ES cells. Among 228 up-regulated miRNAs in five ES cells, the expression of miR-181c was increased by 2.85- to 5.57-fold in comparison with hMSCs.

### Decrease in the expression of FAS in ES cells

Next, the expression profiles of mRNAs in ES cell lines were analyzed using cDNA array. The data demonstrated that 3043 mRNAs in ES cells exhibited significantly different expression from those in hMSCs. The expressions of 1062 mRNAs out of 3043 significantly increased, whereas those of 1884 were significantly decreased in all ES cell lines tested. The remaining 97 mRNAs showed different expression patterns among five ES cells. Among 1884 down-regulated mRNAs in five ES cells, the expression of FAS (Fig. 1b) was decreased by 2.06- to 24.92-folds in comparison with hMSCs.

### FAS as a direct target of miR-181c in ES cells

**Fig. 2:**
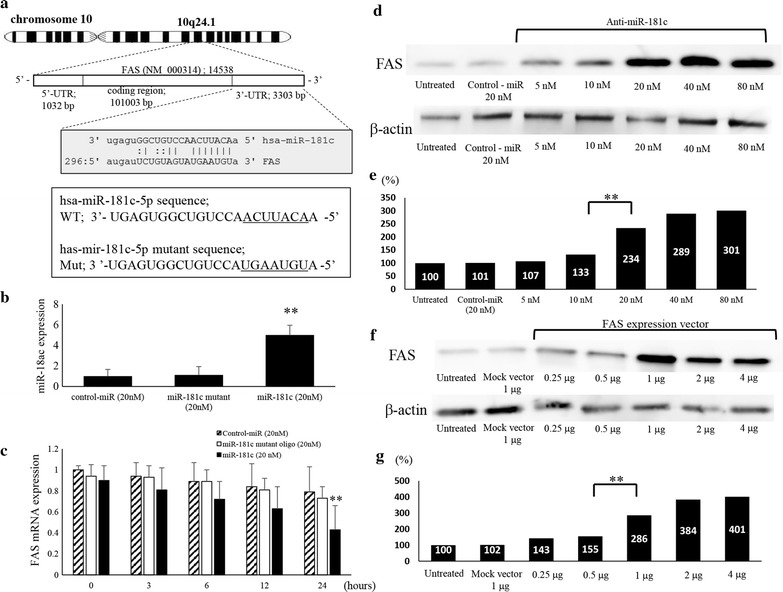
Inhibition of FAS mRNA expression in SKES1 cells. **a** Possible binding sites of miR-181c at the 3′UTR of FAS mRNA. Each sequence of miR-181c (Wt) and its mutant (Mut). **b**, **c** After actinomycin D administration, the miR-181c and mRNA expression level in the negative control-miR, miR-181c, and miR-181c mutant was evaluated by qRT-PCR. *p < 0.05, **p < 0.01. **d** Western blot analysis showed an increase in FAS protein levels upon anti-miR-181c and FAS-expression vector treatment. **e** Densitometric evaluation of FAS protein. *p < 0.05, **p < 0.01. **f** Western blot showing increase in the expression of FAS protein by the transfection of FAS expression vector in SKES1 cells. **g** The expression of FAS protein after induction of FAS vector. **p < 0.01

The BLAST and TargetScan analyses demonstrated a considerable complementarity in the sequence of miR-181c seed region with human FAS mRNA 3′un-translated region (3′-UTR) (Fig. 2a) suggesting the influence of miR-181c to FAS mRNA via association with 3′UTR of the mRNA. Therefore, we examined the effects of miR-181c on the expression of FAS in ES cells by the transfection of miR-181c and a mutated miR-181c into SK-ES-1 cells. In this experiment, de novo mRNA transcription was blocked using actinomycin D (10 μg/ml), an inhibitor of mRNA transcription, since we attempted to determine whether FAS mRNA stability would be affected by miR-181c. Using a microRNA mutant oligonucleotide method instead of the luciferase method, we have provided evidence that the microRNA in question disrupts and/or interferes with expression of the target mRNA [11–13]. We observed an increased intracellular miR-181c level by 5.01 ± 0.94 folds compared with control-miR (Fig. 2b) and significantly decreased FAS expression by 0.43 ± 0.23 folds at mRNA level after the transfection with miR-181c oligonucleotide (Fig. 2c). The miR-181c transfected cells increased 5.01 times, which is the combined total of endogenous miR-181c and transfected oligo, but we have not analyzed the exact proportion of endogenous miR-181c. This result is regarded as verification to confirm that miR-181c oligonucleotides can be properly transfected into the cell. The results suggested that the stability of FAS mRNA was inhibited by miR-181c in ES cell lines.

### Effects of anti-miR-181c on the expression of FAS

We next examined the effects of anti-miR-181c oligonucleotide, an inhibitor of miR-181c, on the expression of FAS in SK-ES-1 cells. In anti-miR-181c transfected cells, the levels of FAS protein remarkably elevated in comparison with untreated or control oligonucleotide-treated cells (Fig. 2d). The level of FAS protein expression in the cells transfected with anti-miR-181c (20 nM) was up-regulated to 2.34-fold of that in the control cells (*p *< 0.01) (Fig. 2e). The western blot analyses further demonstrated that the levels of FAS protein in FAS expression vector transfected cells was significantly increased than those in Mock vector transfected cells (Fig. 2f). In comparison with untreated cells, the cells transfected FAS vector (1 μg) showed significant increase in the expression of FAS protein to 2.86-fold (*p *< 0.01) (Fig. 2g).

### Inhibition of ES cell proliferation by anti-181c-miR and FAS

**Fig. 3:**
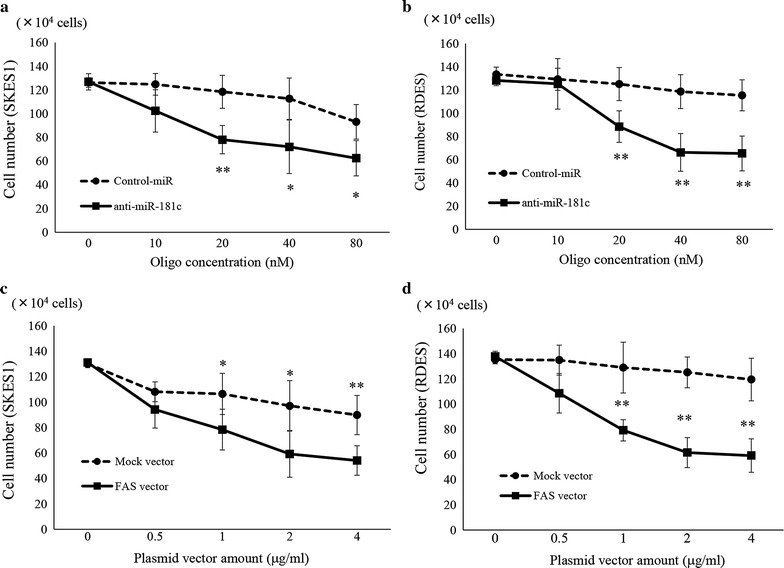
Cell growth assay to study the effects of anti-miR-181c. **a**, **b** Anti-miR-181c inhibits cell growth in SKES1 cells, and RDES cells. **c**, **d** FAS expression vector inhibits cell growth in SKES1 cells and RDES cells. Data represents mean ± SD from three independent experiments. Differences with p < 0.05 were considered statistically significant. *p < 0.05, **p < 0.01

To examine the effects of FAS on the cell proliferation of ES, FAS expression vector was transfected into SK-ES-1 cells. Since the introduction of anti-miR-181c lead to the increase in the expression of FAS, we also investigated the effects of anti-miR-181c on the growth of ES cells. Compared to negative control-miRNA-transfected cells (118.5 ± 13.9 × 10^5^ cells), anti-miR-181c (20 nM) transfected SK-ES-1 cells showed a significant decrease in the cell number (78.1 ± 11.9 × 10^5^ cells) at 48 h after transfection (p = 0.0083). Treatment with anti-miR-181c (40 nM) also inhibited the proliferation of SK-ES-1 cells (72.1 ± 22.5 × 10^5^ cells) compared to in negative control-miRNA-transfected cells (112.7 ± 17.5 × 10^5^ cells) (p = 0.031). The cell growth of SK-ES-1 (62.5 ± 14.9 × 10^5^ cells) was inhibited by the transfection of anti-miR-181c (80 nM) as determined by cell counting in comparison with negative control-miRNA transfected cells (93.1 ± 14.6 × 10^5^ cells) (p = 0.044) (Fig. 3a). The treatment with anti-miR-181c also inhibited the proliferation of RD-ES cells (88.5 ± 13.6 × 10^5^ cells) in comparison with that of NC-miRNA transfected cells (125.2 ± 14.2 × 10^5^ cells) (p < 0.01) (Fig. 3b). The cell growth of SK-ES-1 (78.4 ± 15.9 × 10^5^ cells) was significantly inhibited by the transfection of FAS expression vector (1 μg/ml) as determined by cell counting in comparison with Mock vector transfected cells (106.5 ± 16.2 × 10^5^ cells) (p = 0.033). The cell growth of SK-ES-1 (59.3 ± 18.3 × 10^5^ cells) was significantly inhibited by the transfection of FAS expression vector (2 μg/ml) as determined by cell counting in comparison with Mock vector transfected cells (97.1 ± 19.9 × 10^5^ cells) (p = 0.027). Compared to SK-ES-1 cells transfected with mock vector (89.9 ± 15.4 × 10^5^ cells), cell growth was significantly decreased in SK-ES-1 cells transfected with the FAS expression vector (4 μg/ml) (54.1 ± 11.6 × 10^5^ cells) (p = 0.0064) (Fig. 3c). Furthermore, in comparison with RD-ES cells transfected by mock vector (79.2 ± 2.22 × 10^5^ cells), the cell growth was significantly decreased in RD-ES cells transfected by FAS expression vector (129 ± 20.2 × 10^5^ cells) (p < 0.01) (Fig. 3d).

### Apoptosis induction by anti-miR-181c and FAS

**Fig. 4:**
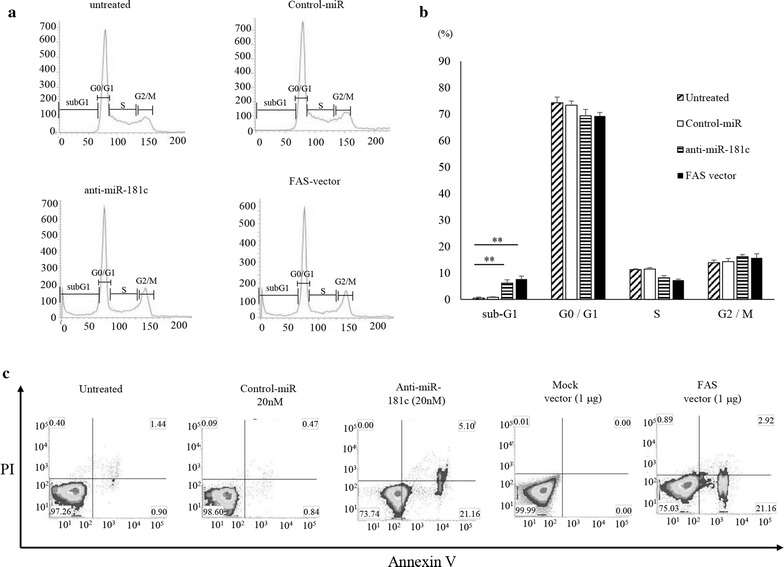
Treatment induces cell cycle retardation. **a** Cells were untreated or transfected with negative control miR, anti-miR-181c or FAS expression vector and stained with propidium iodine and analyzed for cell cycle distribution. *p < 0.05, **p < 0.01. **b** Histograms shown are the mean values of each cell cycle phase: *p < 0.05, **p < 0.01. **c** SKES1 cells treated with Anti-miR-181c and FAS expression vector were stained with Annexin V-FITC/PI, and analyzed for cell apoptosis distribution

In order to identify whether the observed inhibition of growth of ES cells was mediated by cell cycle retardation or apoptosis induction, we analyzed the cell cycle distribution of anti-miR-181c or FAS expression vector treated cells (Fig. 4a). FACS analysis revealed that the number of SK-ES-1 cells transfected with anti-miR-181c (6.2 ± 1.2%) or FAS vector (7.7 ± 1.1%) in sub-G1 phase was significantly higher than that in untreated (0.5 ± 0.4%) and negative control (0.8 ± 0.2%) transfected cells. Whereas that in G0/G1 or G2/M phase showed no significant differences compared with untreated and negative control transfected cells (Fig. 4b). The results indicated that anti-miR-181c and FAS might have no effect on the cell cycle progression of ES cells. Double staining with Annexin V-FITC and PI further demonstrated the induction of apoptosis in SK-ES-1 cells transfected with anti-miR-181c or FAS vector compared with untreated and negative control transfected cells (Fig. 4c). The anti-miR-181c and FAS vector transfected groups had increased sub-G1 fraction compared to the untreated and control-miR groups, indicating that apoptosis had been induced. However, there was no difference in the proportion of the G0/G1, S, G2/M divisions among the 4 groups, so transfection of the anti-miR-181c and FAS vector had no effect on the cell cycle.

### FAS restoration induced caspase and PARP cleavage

**Fig. 5:**
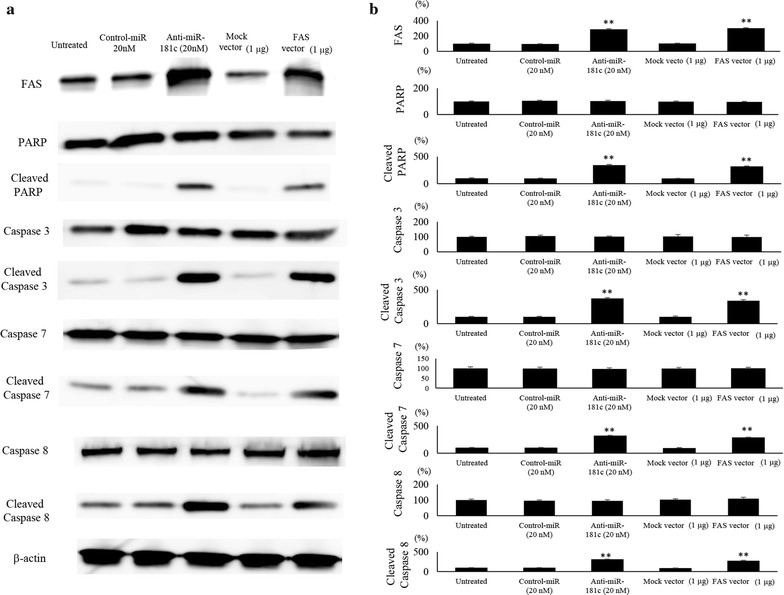
Immunoblot analysis of FAS signaling related factors. **a** FAS and apoptosis related factors to study the effect of anti-miR-181c and FAS vector on FAS related apoptotic pathway in SKES1 cells. **b** The quantification of western blot analysis. Data represents represent the mean ± SD of three independent experiments. p < 0.05 was considered to indicate significance: *p < 0.05, **p < 0.01

To examine the correlation between FAS and cleaved PARP, the expression of FAS, caspase 3/7/8, PARP and their cleaved forms were investigated in ES cells (Fig. 5a). SK-ES-1 cells that were transfected with anti-miR-181c or FAS expression vector showed the increase in expression of FAS, and cleaved caspase 3, 7, and 8 (Fig. 5b). FAS expression in SK-ES-1 cells was increased transfected with the anti-miR-181c (288 ± 2.9%) and FAS vector (303 ± 3.1%) compared to in control cells (100%). When SK-ES-1 cells were transfected with the anti-miR-181c, the expression of cleaved PARP (342 ± 14.1%), cleaved caspase 3 (372 ± 12.2%), cleaved caspase 7 (321 ± 5.95%) and cleaved caspase 8 (315 ± 3.24%) was dramatically increased compared to in untreated SE-ES-1 cells (100%). When SK-ES-1 cells were transfected with the FAS expression vector, the expression of cleaved PARP (319 ± 8.1%), cleaved caspase 3 (337 ± 16.3%), cleaved caspase 7 (289 ± 4%) and cleaved caspase 8 (278 ± 7.8%) was dramatically increased compared to in untreated SK-ES-1 cells (100%).

### Growth inhibition of xenografted tumors by anti-miR-181c and FAS

**Fig. 6:**
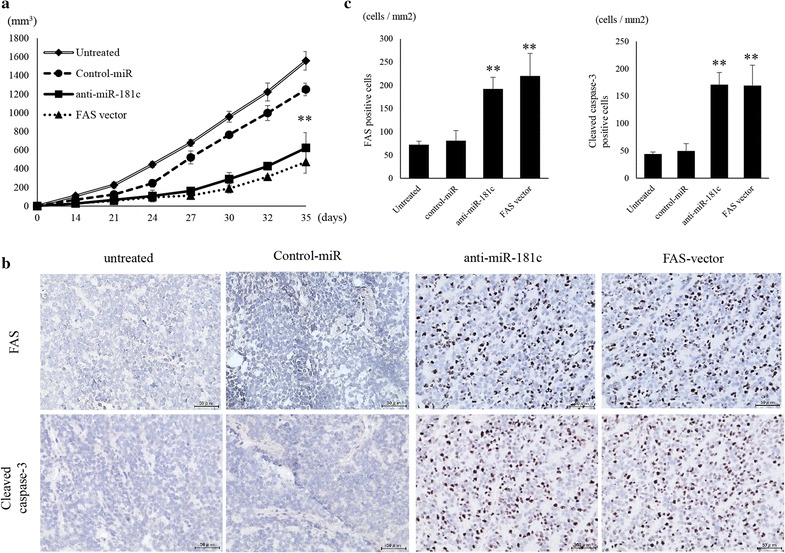
Suppression of ES tumor growth by anti-miR-181c and FAS vector in vivo. **a** Four groups of mice inoculated with (1) untreated SKES1 cells (n = 7); (2) transfected with negative control-miR (n = 7); (3) transfected with anti-miR-181c (n = 7); and (4) transfected with FAS expression vector (n = 7) were generated. Tumor volumes were measured every 3 days during the treatment. **p < 0.01. **b** Immunohistochemistry image analysis of FAS and cleaved caspase 3 following the transfection of anti-miR-181c and FAS vector. **c** The number of FAS and cleaved caspase 3 positive cells per unit area. Data represents represent the mean ± SD of three independent experiments. p < 0.05 was considered to indicate significance: *p < 0.05, **p < 0.01

The effects of miR-181c inhibitor on the growth of xenografts in vivo were examined next. The introduction of anti-miR-181c into SKES1 cells resulted in the decreased growth of subcutaneous xenografted tumors in nude mice (Fig. 6a). The size of tumors in mice inoculated with anti-miR-181c-transfected SK-ES-1 cells (466.5 ± 28.1 cells/mm^3^) or FAS vector-transfected cells (385.5 ± 16.9 cells/mm^3^) was significantly smaller than that with untreated (1165.8 ± 74.1 cells/mm^3^) and NC-miRNA-transfected cells (1021.2 ± 54.7 cells/mm^3^). The results suggested that inhibition of expression of miR-181c also lead to reduction of in vivo growth of ES cells. Immunohistochemical studies revealed that the expression of FAS and cleaved caspase 3 in the xenografted tumors was inhibited by the transfection with anti-miR-181c and FAS expression vector (Fig. 6b). The number of cells positive for FAS expression was significantly increased in mice inoculated with anti-miR-181c (199 ± 21.8 cells/mm^2^) or FAS expression vector transfected cells (230.7 ± 45.7 cells/mm^2^) than that with untreated (65.6 ± 10.6 cells/mm^2^) or control-miR transfected cells (93.8 ± 16.8 cells/mm^2^) (p < 0.01). The number of cleaved caspase 3 expressing cells was significantly increased in mice inoculated with anti-miR-181c (170.9 ± 22.2 cells/mm^2^) or FAS vector (169.3 ± 37.4 cells/mm^2^) transfected cells than that with untreated (44.1 ± 3.6 cells/mm^2^) or control-miR transfected cells (49.7 ± 13.3 cells/mm^2^) (p < 0.01) (Fig. 6c).

## Discussion

MiRNAs are a group of small, noncoding RNAs that regulate the protein coding genes [14, 15]. To recognize consequential miRNA–mRNA relationship in ES, we carried out genome-wide miRNA array and cDNA array. In the present study, miRNA array analysis showed that the expression of miR-181c was upregulated in all of the five tested ES cell lines. Several studies have shown that miR-181c is involved in various biological and pathological processes, including development, differentiation, inflammation, apoptosis, and cancer [5, 16]. Up-regulation of miR-181c is reported to be involved in tumor cell growth [4] and chemotherapy resistance [17] in other malignant tumors.

The data from cDNA array analysis showed that FAS mRNA expression is decreased in all five ES cell lines compared with hMSCs. Furthermore, sequence analysis suggested possible association of miR-181c with 3′UTR of FAS. Numerous reports suggest a key role for the transcriptional regulation by FAS, in the complex signaling network of apoptosis. Malignant tumor cells tend to downregulate FAS expression to avoid FAS-mediated apoptosis signaling [16, 18]. Our data in ES cells is consistent with previous studies reporting that the downregulation of FAS may contribute to malignant phenotypes.

Although miR-181c might influence the expression of many genes, we focused on FAS as the target of miR-181c in ES cells. Our cDNA array analysis demonstrated that FAS was the only miR-181c-target gene whose expression was uniformly upregulated in all five ES cell lines, whereas the expression of other candidate genes was different among the ES cells. Analysis using several algorithms, such as BLAST and TargetScan, further suggested that FAS was a putative target of miR-181c. Among these abnormalities, from a cDNA array, we first focused on FAS as a gene generally considered strongly affect the biology of cancer cells. Also, when we examined whether microRNA that binds to the FAS mRNA 3′-UTR is elevated in a microRNA array, we found that miR-181c was elevated in all five Ewing sarcoma cell lines. Therefore, we hypothesized that an elevation in miR-181c results in inhibition of FAS mRNA expression, and we investigated our hypothesis. Therefore, we analyzed the possibility that miR-181c might play an anti-cancer regulatory role in ES cells by targeting FAS. However, FAS mRNA degradation was promoted even after the termination of de novo mRNA transcription. This effect was not observed in the mutant miR-treated cells. Therefore, miR-181c may have affected FAS mRNA directly at least in part.

We examined the functions of miR-181c in the regulation of its possible target gene, FAS, and the changes in the biological characteristics of ES cell lines. Forced expression of miR-181c resulted in the repression of FAS protein, indicating that miR-181c might function as an oncogene in ES cells. Our results are the first evidence that suggest that the same miR-181c mediated regulatory mechanism of FAS expression might exist in ES cells.

Our data shows that miR-181c promotes the proliferation of ES cells via induction of anti-apoptosis mechanisms, and not by affecting the cell cycle pausing at G1/G0 phase. Transfection of anti-miR-181c or FAS expression vector into SKES1 cells resulted in the increase of sub G1 fraction but did not influence the proportion between G1/G0, S and G2/M phases. Thus, we can infer that the upregulation of FAS, resulting from transfection of anti-miR-181c or FAS expression vector, induced apoptosis of ES cells. This indicates that the repression of ES cell growth with FAS restoration was a result of apoptosis rather than the cell cycle retardation.

Considering that FAS acts as a major regulator of apoptosis related pathways via caspase and PARP cleavage, the repression of FAS by 181c-miR might play an important role in apoptosis of other types of cells as well. The receptor ligation followed by binding with Fas-associated death domain protein leads to the recruitment of caspase-8, resulting in the cleavage and activation of caspase-8 and downstream caspases [19]. Caspase-8 subsequently activates caspase-3 [20]. Our results show that transfection with anti-miR-181c and FAS expression vector enhances caspase-8, and caspase 3/7 activity, and increases cleaved PARP expression levels.

Furthermore, the repression of miR-181c results in the inhibition of ES tumor growth in vivo. Consistent with the data from in vitro experiments, xenograft ES models also indicated that miR-181c repression is capable of inhibiting ES tumor development in vivo following restoration of FAS expression and translation.

## Conclusions

The present study indicates the inverse correlation of miR-181c and FAS in ES cells for the first time. We show that miR-181c regulates FAS and the FAS-mediated apoptosis pathway by directly downregulating FAS expression. These results suggest that miR-181c is a regulator of FAS mediated apoptosis in Ewing’s sarcoma. FAS plays a central role in the physiological regulation of apoptosis, and has been implicated in the pathogenesis of various malignancies [21–23]. Our data suggests that FAS is one of the crucial factors that enhance tumor proliferation in ES, as well as other malignant tumors. Although the data demonstrated in the present study needs to be confirmed using clinical samples of ES, the novel information regarding the link between miR-181c and FAS in ES cells would be beneficial for the better understanding of oncogenesis of ES and provide novel strategies for clinical application in the future.

## Abbreviations

ES: Ewing’s sarcoma
hMSCs: human mesenchymal stem cells
miRNAs: microRNAs
FACS: fluorescence activated cell sorting
NC: negative control
PI: propidium iodide
ANOVA: analysis of variance
qRT-PCR: quantificational real-time polymerase chain reaction
